# Supplementary material for: Pyruvate Dehydrogenase Kinase 1 inhibition mediated oxidative phosphorylation enhancement in cartilage promotes osteoarthritis progression
Source: BMC Musculoskelet Disord. 2023 Jul 20;24:597. doi: 10.1186/s12891-023-06585-6 (PMC10357736; doi:10.1186/s12891-023-06585-6)
Supplement: Supplementary file 1 — Additional file 1. [file 12891_2023_6585_MOESM1_ESM.zip › BP.Gsea.1653623667859/GOBP_ESTABLISHMENT_OF_PROTEIN_LOCALIZATION_TO_ENDOPLASMIC_RETICULUM.html]

Details for gene set GOBP\_ESTABLISHMENT\_OF\_PROTEIN\_LOCALIZATION\_TO\_ENDOPLASMIC\_RETICULUM[GSEA]

|  || Dataset | 55457\_collapsed\_to\_symbols.cls.cls #OA\_versus\_normal.cls.cls #OA\_versus\_normal\_repos |
| Phenotype | cls.cls#OA\_versus\_normal\_repos |
| Upregulated in class | OA |
| GeneSet | GOBP\_ESTABLISHMENT\_OF\_PROTEIN\_LOCALIZATION\_TO\_ENDOPLASMIC\_RETICULUM |
| Enrichment Score (ES) | 0.419414 |
| Normalized Enrichment Score (NES) | 1.5412579 |
| Nominal p-value | 0.024886878 |
| FDR q-value | 0.31064883 |
| FWER p-Value | 1.0 |
Table: GSEA Results Summary

  

Fig 1: Enrichment plot: GOBP\_ESTABLISHMENT\_OF\_PROTEIN\_LOCALIZATION\_TO\_ENDOPLASMIC\_RETICULUM      
 Profile of the Running ES Score & Positions of GeneSet Members on the Rank Ordered List

  

| SYMBOL | TITLE | RANK IN GENE LIST | RANK METRIC SCORE | RUNNING ES | CORE ENRICHMENT || 1 | SPCS1 | signal peptidase complex subunit 1 [Source:HGNC Symbol;Acc:HGNC:23401] | 303 | 0.747 | 0.0581 | Yes |
| 2 | UBL4A | ubiquitin like 4A [Source:HGNC Symbol;Acc:HGNC:12505] | 464 | 0.675 | 0.1194 | Yes |
| 3 | CHMP4A | charged multivesicular body protein 4A [Source:HGNC Symbol;Acc:HGNC:20274] | 526 | 0.649 | 0.1857 | Yes |
| 4 | TRAM1 | translocation associated membrane protein 1 [Source:HGNC Symbol;Acc:HGNC:20568] | 569 | 0.633 | 0.2516 | Yes |
| 5 | RYR2 | ryanodine receptor 2 [Source:HGNC Symbol;Acc:HGNC:10484] | 828 | 0.561 | 0.2928 | Yes |
| 6 | GET3 | "guided entry of tail-anchored proteins factor 3, ATPase [Source:HGNC Symbol;Acc:HGNC:752]" | 1161 | 0.485 | 0.3200 | Yes |
| 7 | SEC61G | SEC61 translocon subunit gamma [Source:HGNC Symbol;Acc:HGNC:18277] | 1163 | 0.485 | 0.3729 | Yes |
| 8 | SEC62 | "SEC62 homolog, preprotein translocation factor [Source:HGNC Symbol;Acc:HGNC:11846]" | 1272 | 0.464 | 0.4152 | Yes |
| 9 | RAB3GAP1 | RAB3 GTPase activating protein catalytic subunit 1 [Source:HGNC Symbol;Acc:HGNC:17063] | 1766 | 0.389 | 0.4194 | Yes |
| 10 | SPCS3 | signal peptidase complex subunit 3 [Source:HGNC Symbol;Acc:HGNC:26212] | 2772 | 0.273 | 0.3712 | No |
| 11 | TRAM2 | translocation associated membrane protein 2 [Source:HGNC Symbol;Acc:HGNC:16855] | 4913 | 0.079 | 0.2135 | No |
| 12 | BAG6 | BAG cochaperone 6 [Source:HGNC Symbol;Acc:HGNC:13919] | 5023 | 0.071 | 0.2128 | No |
| 13 | SEC61A1 | SEC61 translocon subunit alpha 1 [Source:HGNC Symbol;Acc:HGNC:18276] | 5378 | 0.041 | 0.1898 | No |
| 14 | SRP9 | signal recognition particle 9 [Source:HGNC Symbol;Acc:HGNC:11304] | 5498 | 0.033 | 0.1842 | No |
| 15 | EDEM1 | ER degradation enhancing alpha-mannosidase like protein 1 [Source:HGNC Symbol;Acc:HGNC:18967] | 5633 | 0.021 | 0.1761 | No |
| 16 | SSR3 | signal sequence receptor subunit 3 [Source:HGNC Symbol;Acc:HGNC:11325] | 5969 | -0.002 | 0.1503 | No |
| 17 | HERPUD1 | homocysteine inducible ER protein with ubiquitin like domain 1 [Source:HGNC Symbol;Acc:HGNC:13744] | 6520 | -0.044 | 0.1123 | No |
| 18 | SRPRA | SRP receptor subunit alpha [Source:HGNC Symbol;Acc:HGNC:11307] | 6566 | -0.047 | 0.1140 | No |
| 19 | SRP19 | signal recognition particle 19 [Source:HGNC Symbol;Acc:HGNC:11300] | 6945 | -0.075 | 0.0928 | No |
| 20 | SPCS2 | signal peptidase complex subunit 2 [Source:HGNC Symbol;Acc:HGNC:28962] | 6981 | -0.078 | 0.0987 | No |
| 21 | SRPRB | SRP receptor subunit beta [Source:HGNC Symbol;Acc:HGNC:24085] | 7553 | -0.123 | 0.0678 | No |
| 22 | RAB3GAP2 | RAB3 GTPase activating non-catalytic protein subunit 2 [Source:HGNC Symbol;Acc:HGNC:17168] | 7957 | -0.152 | 0.0531 | No |
| 23 | SEC63 | "SEC63 homolog, protein translocation regulator [Source:HGNC Symbol;Acc:HGNC:21082]" | 8151 | -0.167 | 0.0563 | No |
| 24 | SRP72 | signal recognition particle 72 [Source:HGNC Symbol;Acc:HGNC:11303] | 8338 | -0.181 | 0.0617 | No |
| 25 | SEC61B | SEC61 translocon subunit beta [Source:HGNC Symbol;Acc:HGNC:16993] | 8510 | -0.194 | 0.0696 | No |
| 26 | SRP14 | signal recognition particle 14 [Source:HGNC Symbol;Acc:HGNC:11299] | 9288 | -0.255 | 0.0371 | No |
| 27 | MAN1A1 | mannosidase alpha class 1A member 1 [Source:HGNC Symbol;Acc:HGNC:6821] | 10207 | -0.341 | 0.0031 | No |
| 28 | HSPA5 | heat shock protein family A (Hsp70) member 5 [Source:HGNC Symbol;Acc:HGNC:5238] | 10820 | -0.407 | 0.0001 | No |
| 29 | SGTA | small glutamine rich tetratricopeptide repeat co-chaperone alpha [Source:HGNC Symbol;Acc:HGNC:10819] | 11185 | -0.455 | 0.0216 | No |
| 30 | SEC61A2 | SEC61 translocon subunit alpha 2 [Source:HGNC Symbol;Acc:HGNC:17702] | 11352 | -0.479 | 0.0610 | No |
| 31 | SRP54 | signal recognition particle 54 [Source:HGNC Symbol;Acc:HGNC:11301] | 11754 | -0.541 | 0.0890 | No |
Table: GSEA details [plain text format]

  

Fig 2: GOBP\_ESTABLISHMENT\_OF\_PROTEIN\_LOCALIZATION\_TO\_ENDOPLASMIC\_RETICULUM      
 Blue-Pink O' Gram in the Space of the Analyzed GeneSet

  

Fig 3: GOBP\_ESTABLISHMENT\_OF\_PROTEIN\_LOCALIZATION\_TO\_ENDOPLASMIC\_RETICULUM: Random ES distribution      
 Gene set null distribution of ES for **GOBP\_ESTABLISHMENT\_OF\_PROTEIN\_LOCALIZATION\_TO\_ENDOPLASMIC\_RETICULUM**
